# The Expression Characteristics of NPF Genes and Their Response to Vernalization and Nitrogen Deficiency in Rapeseed

**DOI:** 10.3390/ijms22094944

**Published:** 2021-05-06

**Authors:** Hongbo Chao, Jianjie He, Qianqian Cai, Weiguo Zhao, Hong Fu, Yingpeng Hua, Maoteng Li, Jinyong Huang

**Affiliations:** 1 School of Agricultural Sciences, Zhengzhou University, Zhengzhou 450001, China; chaohongbo@zzu.edu.cn (H.C.); caiqianqianhn@163.com (Q.C.); 15290885289@163.com (H.F.); yingpenghua@zzu.edu.cn (Y.H.); 2 Department of Biotechnology, College of Life Science and Technology, Huazhong University of Science and Technology, Wuhan 430074, China; hejianjie@hust.edu.cn (J.H.); zhaoweiguo0517@126.com (W.Z.); 3 Hybrid Rape Research Center of Shaanxi Province, Yangling 712100, China

**Keywords:** *Brassica napus*, *NPF* gene, transfer protein, gene expression, growth and development

## Abstract

The *NITRATE TRANSPORTER 1/PEPTIDE TRANSPORTER FAMILY* (*NPF*) genes, initially characterized as nitrate or peptide transporters in plants, are involved in the transport of a large variety of substrates, including amino acids, nitrate, auxin (IAA), jasmonates (JAs), abscisic acid (ABA) and gibberellins (GAs) and glucosinolates. A total of 169 potential functional *NPF* genes were excavated in *Brassica napus*, and they showed diversified expression patterns in 90 different organs or tissues based on transcriptome profile data. The complex time-serial expression changes were found for most functional *NPF* genes in the development process of leaves, silique walls and seeds, which indicated that the expression of *Brassica napus NPF* (*BnaNPF*) genes may respond to altered phytohormone and secondary metabolite content through combining with promoter element enrichment analysis. Furthermore, many *BnaNPF* genes were detected to respond to vernalization with two different patterns, and 20 *BnaNPF* genes responded to nitrate deficiency. These results will provide useful information for further investigation of the biological function of *BnaNPF* genes for growth and development in rapeseed.

## 1. Introduction

Plant *NPF* (*NITRATE TRANSPORTER 1/PEPTIDE TRANSPORTER FAMILY*) proteins display sequence homology with the proton-coupled oligopeptide transporter (POT) family of peptide transporters, belong to the large peptide transporter (PTR) family [1] and are involved in dietary nitrogen absorption in the form of di- and tripeptides [2,3]. In plants, *NPF* members are initially characterized as nitrate or peptide transporters. At*NPF*6.3, known as AtNRT1.1, is the first plant member discovered as a nitrate transporter in *Arabidopsis* [4], and the crystal structure of *AtNPF*6.3 recently determined showed the similarity with PTRs in the bacterial [5,6]. Subsequently, some *NPF* members are identified in different plants and demonstrated that they behave as dipeptide transporters [7,8]. So, nitrate/peptide transport function is believed to be a specific feature of this family in plants over a period of time. However, in recent years, several studies demonstrated that some *NPF* members could transport an even wider range of substrates, including nitrite, chloride, auxin (IAA), abscisic acid (ABA), jasmonates (JAs), and gibberellins (GAs) and glucosinolates [9,10,11,12]. Additionally, some of the *NPF* members are even able to transport more than one different substrate: nitrate/IAA, nitrate/ABA, nitrate/glucosinolates, peptides/amino acids, or GA/JA.

After identifying the first nitrate transporter *NPF*6.3/NRT1.1, Tsay et al. [4,8] subsequently characterized more than half of the *NPF* nitrate transporters. So far, more than 21 *NPF* members have been demonstrated to be able to transport nitrate in *Arabidopsis*, many of which are able to transport other substrates, such as ABA, GA, JA and glucosinolates [8,13]. Screening 45 out of 53 *AtNPF* members using a modified yeast two-hybrid system with ABA, GA and JA-Ile specific receptor complexes, Chiba et al. [14] confirmed that nine *NPF* members had an ABA transport function, 18 *NPF* members were able to transport GA and 13 *NPF* members were able to transport the bioactive JA/JA-Ile. NRT1.1/*NPF*6.3 was confirmed to be able to transport IAA and 2,4-D [15,16]. In addition, PTR3/*NPF*5.2, PTR1/*NPF*8.1, PTR5/*NPF*8.2 and PTR2/NTR1/*NPF*8.3 were identified as dipeptide and tripeptide transporters using complementation of yeast strains deficient for peptide uptake [17,18].

Based on the phylogenetic relationship, *NPF* members were divided into eight subfamilies (from *NPF*1 to *NPF*8) [1]. In the *NPF*1 subfamily, Mt*NPF*1.7, from *Medicago truncatula*, has been characterized to be involved in nodulation and root architecture and behaves as a high-affinity nitrate transporter [19,20,21]. *NPF*1.1 and *NPF*1.2, from *Arabidopsis*, are confirmed to be important for redistributing xylem-borne nitrate to enhance plant growth [22]. The *NPF*2 subfamily contains nitrate, phytohormone and glucosinolates transporters, and *NPF*2 members show a wide range of tissue and developmental specificity in *Arabidopsis*. At*NPF*2.3, as a root stele transporter, contributes to nitrate translocation to shoots under salt stress [23], and the low-affinity nitrate transporter At*NPF*2.12 is responsible for nitrate-dependent early embryo development [24]. Besides, five At*NPF* members that are capable of transporting glucosinolates belong to the *NPF*2 subfamily [11]. *AtNPF*3.1 is the only member of the *NPF*3 subfamily in *Arabidopsis* and plays the role of nitrite transport [25]. It has been demonstrated that ABA could be transported by *NPF*4 subfamily members [10], and some members of the *NPF*4 subfamily are able to transport GA, such as *NPF*4.1 and *NPF*4.2. *NPF*5 is the largest *NPF* subfamily and contains numerous members involved in nitrate, ABA, GA, JA and di-peptides transport [14,26,27]. In the *NPF*6 subfamily, most of the members have been demonstrated to be nitrate transporters, including the first identified *NPF* member At*NPF*6.3 [4,28,29,30]. At least two different substrates were able to be transported by *NPF*7 subfamily members based on three well functionally characterized proteins: At*NPF*7.2/NTR1.8 and At*NPF*7.3/NTR1.5 for nitrate transport [31,32], as well as Os*NPF*7.3 for dipeptide transport [33]. Three *Arabidopsis NPF*8 subfamily members, At*NPF*8.1/PTR1, At*NPF*8.2/PTR5 and At*NPF*8.3/PTR2, have been proved to be dipeptide transporters, while At*NPF*8.1 and At*NPF*8.2 are able to transport JA-Ile simultaneously [34,35]. It is noteworthy that 38 and 17 *NPF* genes have been characterized at the functional level in *Arabidopsis* and rice, respectively, and more than half of them function on nitrate transport and are distributed on eight *NPF* subfamilies, and play important roles in nitrate absorption, translocation and utilization [13,31,36,37]. Generally, *NPF* transporters with a very broad substrate specificity have an important function in plant growth and development, and genome-wide identification has been implemented in poplar [38], alfalfa [36], apple [39] and wheat [13], but a few systematic analyses have been conducted for *NPF* genes in *Brassica* species.

*B. napus*, an allotetraploid evolved from the hybridization between two diploid progenitors (*Brassica rapa* and *Brassica oleracea*), is an important oil crop in the world. Compared with *Arabidopsis*, *B. napus* has experienced a whole-genome triplication, which occurred between 7.9 and 14.6 million years ago [40], and a hybridization event via the natural crossing of *B. rapa* and *B. oleracea* (both of which diverged from a common ancestor ~\n4 million years ago) about 7500 years ago [41,42,43]. Recently, the genomes of ‘Darmor-*bzh*’ and ‘ZS11’ have been successfully sequenced and assembled [42,44], and Pan-genomes have been constructed based on next-generation sequencing technologies for *B. napus*, which facilitate systematically excavating *NPF* genes in rapeseed. Although the NPF genes have been identified in rapeseed [45,46], functional NPF proteins and genes should be excavated, and their expression characteristics and role in the tissues that play an important role in rapeseed oil yield should be further analyzed. In this study, 169 *NPF* genes were excavated in the *B. napus*, and the expression profiles of *BnaNPF* genes in diverse tissues, as well as time-serial expression changes in leaf, silique wall and seed, were determined; meanwhile, expression changes induced by vernalization at different development stages and response to nitrate deficiency were analyzed. These results will provide useful information for further investigation of the biological function of *BnaNPF* genes for growth and development in *B. napus*.

## 2. Results

### 2.1. Distribution and Synteny Analysis of NPF Genes in Four Brassica Species

**Figure 1:**
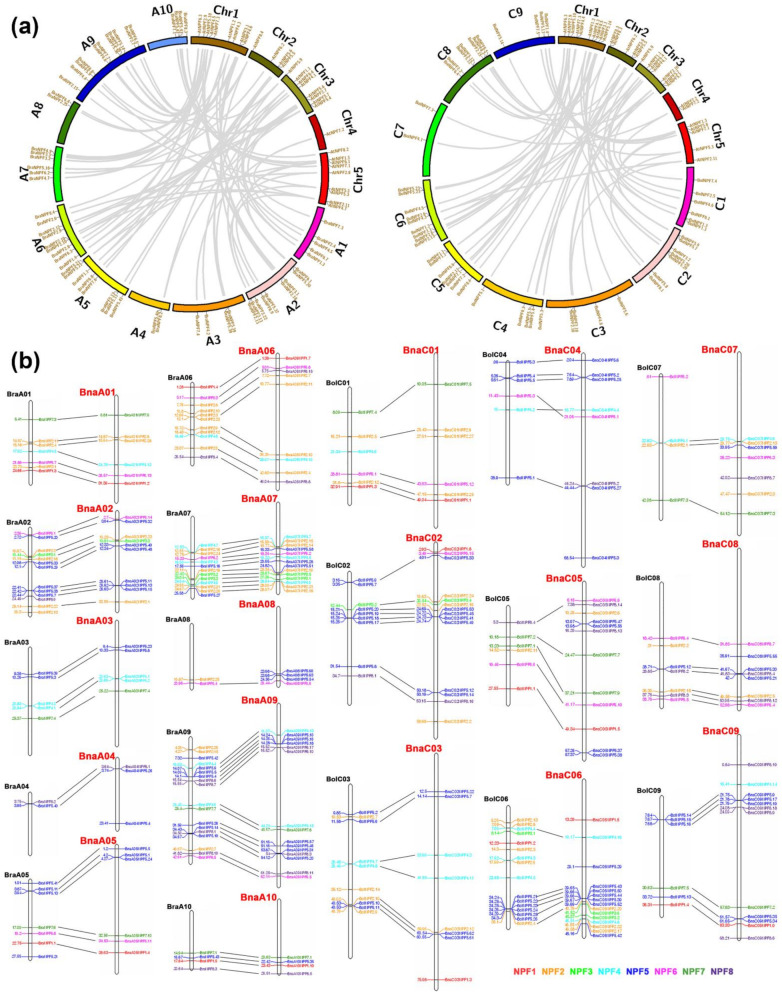
Genomic distribution of the *NPF* genes in *Arabidopsis*, *B. rapa*, *B. oleracea* and *B. napus*, and synteny of the *NPF* genes in the four Brassica species genomes. (**a**) The synteny of *NPF* genes between *Arabidopsis* and *B. rapa* (**Left**), and between *Arabidopsis* and *B. oleracea* (**Right**). (**b**) The collinearity of NPF genes between *B. rapa* and the A sub-genome of *B. napus*, and between *B. oleracea* and the C sub-genome of *B. napus*. Three letters before the chromosome were used to distinguish the species, and the color of the font on the bottom right distinguishes the subfamilies.

Based on BLASTP using 53 *Arabidopsis* NPF protein and phylogenetic analysis (Figure S1), a total of 169 NPF genes encoding 186 proteins were identified in the *B. napus* genome. To investigate the evolution of *BnaNPF* genes, the synteny of *NPF* gene pairs between the *B. rapa* and *Arabidopsis* genome, *B. oleracea* and *Arabidopsis* genome, *B. napus* and *B. rapa* genome, and the *B. napus* and *B. oleracea* was performed to further understand the expansion mechanism of *NPF* genes in *B. napus* (Figure 1). The result shows that most of the *BnaNPF* genes exhibited evolutionary and syntenic relationships with *NPF* genes in *Arabidopsis*, *B. rapa,* and *B. olereaca* (Figure S2), suggesting the contribution to the evolution of the *BnaNPF* gene family. Furthermore, the Ka (nonsynonymous nucleotide substitution rate), Ks (synonymous nucleotide substitution rate) and Ka/Ks (Ka/Ks ratio) of orthologous pairs on *BnaNPF* and At*NPF* genes were calculated to test the evolutionary selection pressure (Table S2). The majority of orthologous *BnaNPF* gene pairs had Ka/Ks < 1, which suggested that most of the *BnaNPF* genes have undergone purifying selection to preserve gene function. The mean value of *NPF*3 (Ka/Ks = 0.10), *NPF*6 (Ka/Ks = 0.11) and *NPF*7 (Ka/Ks = 0.13) gene pairs was lower than other subfamilies, showing that these three subfamilies may have suffered robust purifying selective pressure during evolution. However, some of the *BnaNPF* genes had Ka/Ks > 1, including *BnaA01NPF2.8*, *BnaC01NPF2.9*, *BnaA06NPF2.10*, *BnaC03NPF2.12* and *BnaC01NPF2.25* in the *NPF*2 subfamily, *BnaA09NPF4.15* in the *NPF*4 subfamily, *BnaA05NPF5.1*, *BnaC04NPF5.3*, *BnaC03NPF5.7*, *BnaA03NPF5.8*, *BnaA02NPF5.15*, *BnaA02NPF5.40*, *BnaC02NPF5.41* and *BnaC06NPF5.42* in the *NPF*5 subfamily, and *BnaC09NPF8.19* in the *NPF*8 subfamily, suggesting that these *BnaNPF* genes are subjected to positive selection during the evolution from *Arabidopsis* to rapeseed.

The distribution and synteny of *NPF* genes were marked on the chromosomes of *B. rapa*, *B. oleracea* and *B. napus* (Figure 1b). *NPF* genes are unevenly distributed on every chromosome, and often organized as clusters in the genome of *B. rapa*, *B. oleracea* and *B. napus*. In the *B. napus* genome, the chromosomes A09 and C06 possess the most *BnaNPF* genes (15, respectively), and A08 possess only four *BnaNPF* genes, which were clustered on the chromosome terminal. *NPF* genes distributed on the *B. rapa* and *B. oleracea* genome keep good collinearity with *NPF* genes on the A and C sub-genome of *B. napus*, respectively. *B. rapa* genome contains 82 *NPF* genes, and the corresponding A sub-genome of *B. napus* contains only 76 *NPF* genes; the *B. oleracea* genome contains 70 *NPF* genes, and the corresponding C sub-genome of *B. napus* contains 93 *NPF* genes, which indicates that parts of *NPF* genes from *B. rapa* genome were lost or recombined to the C genome of *B. napus* in the evolution process. For example, *BraNPF5.21* on the terminal of the chromosome BraA05 was replicated and recombined to BnaC05 chromosome (*BnaC05NPF5.37* and *BnaC05NPF5.38*). According to the synteny analysis, 97 *BnaNPF* genes evolved from the *B. rapa* genome, and 72 *BnaNPF* genes from the *B. oleracea* genome. Furthermore, 73 *BraNPF* genes retained synteny with *NPF* genes in the *B. napus* genome, including 55 *BraNPF* genes with a 1:1 synteny relationship, 16 *BraNPF* genes with a 1:2 relationship (duplication in *B. napus* genome) and even two *BraNPF* genes with more than a 1:2 relationship (1:3 and 1:5) (Table 1 and Table S3). Nine *BraNPF* orthologs were not identified in the *B. napus* genome (1:0 relationship) and two *BnaNPF* orthologs were not identified in the *B. rapa* genome (0:1 relationship), suggesting a loss of the gene during evolution. Sixty-one *BolNPF* genes retained synteny with *NPF* genes in the *B. napus* genome, including 54 with a 1:1 relationship and 7 with a 1:2 relationship. Twenty-six and five translocations were identified for *NPF* genes when comparing the *B. napus* genome with the *B. rapa* and *B. oleracea* genome, respectively. Besides, because the genomic data of *B. napus* have not yet been fully mapped to the chromosome, the chromosomal location and evolution of three *BnaNPF* genes (*BnaNPF2.26*, *BnaNPF2.29* and *BnaNPF2.30*) is still unclear.

### 2.2. B. napus Genome Possessed the Most NPF Genes

Using the sequences of 53 *Arabidopsis* NPF family protein as queries to perform BLASTp and the information from the article Leran et al. [1] reported, NPF proteins from 36 sequenced species were retrieved, including *B. rapa, B. oleracea* and *B. napus*. Information regarding genome size and number of *NPF* genes is shown in Table 2. The genome sizes of these 34 plant species ranged from 127.42 Mb (*Arabidopsis*) to 2271.03 Mb (*Zea mays*), and the *NPF* gene number varied from 23 (*Physcomitrella patens*) to 169 (*B. napus*). The *B. napus* genome possessed the most *NPF* genes (167), though its genome size was smaller than that of *Malus domestica* and *Zea mays*, which indicated that the copy number variations of *B. napus NPF* genes might be attributed to their requirement for (un)specific substrates as a result of evolutionary selection, such as some *NPF2* members for transporting glucosinolates [8]. All *NPF* genes were grouped into eight clades with known 53 *NPF* members from *Arabidopsis*. Most plants have more *NPF2, NPF4 and NPF5* subfamily members. *NPF*1 and *NPF*2 subfamilies are absent from the two lower plants *Physcomitrella patens* and *Selaginella moellendorffii*. In addition, based on the BnPIR database that provides more detailed annotation for *B. napus* genes, 11 *BnaNFP* genes were identified to encode two proteins derived from two different transcripts, and three *BnaNFP* genes encode three proteins translated from three different transcripts. Therefore, a total of 186 BnaNPF proteins were identified in *B. napus*, including 17 proteins from different transcripts. Based on the phylogenetic tree (Figure S1), the evolutionary relationship of *NPF* proteins between *B. napus* and *Arabidopsis* was easy to compare and provided a good guide for studying the function of *NPF* genes in *B. napus*. According to known *Arabidopsis NPF* protein subfamily information and phylogenetic tree branches, eight unambiguous clades that represented eight *B. napus NPF* subfamilies were identified. The BnaNPF5 subfamily was the largest because of a larger number of *Arabidopsis* NPF5 members and possessed 63 members (more than a third of the total number of *BnaNPF* genes), followed by NPF2 (30), NPF8 (19), NPF4 (16), NPF6 (15), NPF7 (10), NPF1 (10), and NPF3 (6). Additionally, BnaA05NPF5.1 and BnaC04NPF5.2, located in the same branch with AtNPF5.1, were grouped into the NPF2 clade, which suggested that the two *NPF* genes might be more closely related to *B. napus* NPF2 in evolution. Similarly, BnaA02*NPF*6.14 and BnaC02*NPF*6.15 seemed to be more closely related to NPF7. Most of the phylogenetic branches within the same clade showed a high bootstrap value (>0.80), which reflected the low genetic differentiation of *Arabidopsis* and *B. napus NPF* genes within the subfamily.

### 2.3. BnaNPF Gene Owning the PTR2 Functional Domain Might Be Regulated by Multiple Phytohormones

The gene structures (number and organization exon–intron) are typical evolutionary imprints within certain gene families and are closely related to their function. The exon/intron arrangements of 169 *BnaNPF* genes were analyzed together with 53 At*NPF* by comparing CDS and the corresponding genomic DNA sequences within and between subgroups based on the phylogenetic tree (Figure S3). The *BnaNPF* genes have a higher degree of divergence among gene structure than *NPF* genes in *Arabidopsis* and contained the numbers of exons varying from 2 to 18. *BnaC02NPF1.8* and *BnaC09NPF1.9* in the *NPF*1 subfamily and *BnaC05NPF2.6*, *BnaA06NPF2.7* and *BnaA06NPF6.8* in the *BnaNPF*2 subfamily were significantly longer than other genes and contained the most exons (16, 16, 18, 18 and 8, respectively); however, most of the *BnaNPF* genes contained no more than six exons. *BnaNPF* genes in different branches exhibited different gene structural features, while the genes in the same branch generally had similar intron/exon distribution patterns. For instance, *BnaA05NPF1.4*, *BnaC05NPF1.5* and *BnaA06NPF1.7* in the *BnaNPF*1 subgroup, *BnaC05NPF5.56*, *BnaA09NPF5.57*, *BnaA07NPF5.58* and *BnaC07NPF5.59* in the *BnaNPF*5 subgroup, and *BnaC07NPF7.3*, *BnaA03NPF7.4*, *BnaC01NPF7.5* and *BnaA01NPF7.6* in the *BnaNPF*7 subgroup had almost the same exon/intron distribution characteristics, and different distribution patterns were found between subgroups. To further explore the specific and conserved regions of 186 BnaNPF proteins, four conserved domains, PTR2, MFS_1 (Major facilitator family), Chorismate_bind and PDDEXK_6 (PDDEXK-like family of unknown function), were identified by the HMMER (biosequence analysis using profile hidden Markov models) website (Figure S3). PTR2 domain, responsible for proton-dependent transport, is the signature domain of NPF protein and could be found in each BnaNPF member, suggesting functional conservation. The major facilitator superfamily MFS_1 domain feature was detected to partially overlap or exist within the PTR2 domain in some BnaNPF members (45/186). The chorismite_bind domain involved in chorismate-utilizing was found in BnaC05NPF2.6 and BnaA06NPF2.7, and BnaC03NPF4.4 contained an unknown function PDDEXK_6 domain.

**Figure 2:**
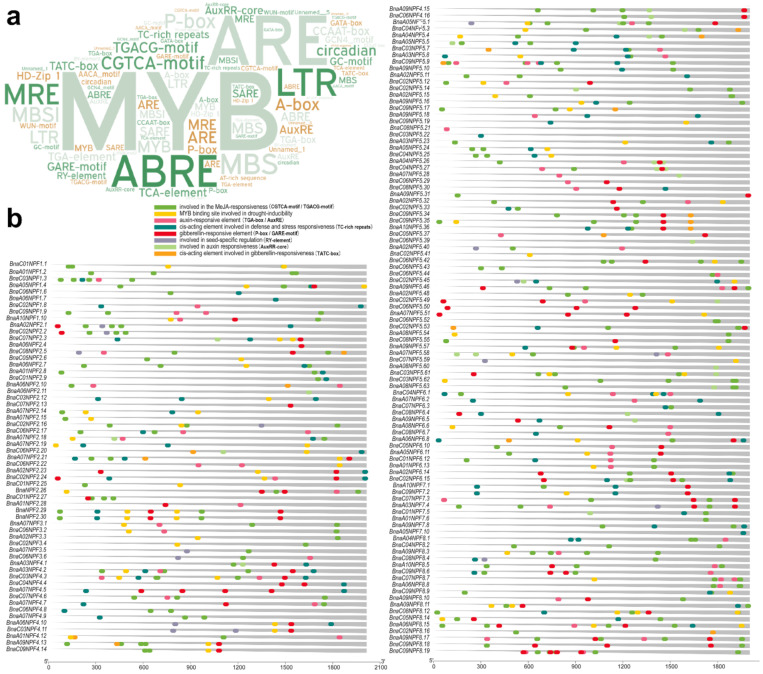
Identification of the CREs of the *BnaNPF* genes. (**a**) Over-presentation of the CREs in the promoters of the 157 *BnaNPF* genes. The bigger the font size, the more frequent the CRE appears in *BnaNPF* genes. (**b**) Genomic distribution and relative abundance of the 8 kinds of CREs involved in the molecular response of plants to phytohormone, abiotic stress responsiveness and seed-specific regulation in the *BnaNPF* gene promoters. Different kinds of CREs are indicated with different colors.

Transcription factors bind to CREs in the promoter and regulate the expression of the target genes [47]. Generally, genes with similar CREs show the same expression patterns. The 2.0-kb upstream regulatory regions of the *BnaNPF* genes were used to explore the CREs (Figure 2 and Table S4). The results show that 157 *BnaNPF* genes contained at least one type of CRE in the promoter regions, which indicated that complex transcriptional regulation might be implicated for *BnaNPF* genes. Apart from the common CREs, such as the CAAT-box, TATA-box and some light-responsive elements (G-box, Box 4, GT1-motif and TCT-motif), some phytohormone-responsive elements, such as the auxin-responsive elements (TGA-element, AuxRR-core, GATA-box, TGA-box and AuxRE), the ABA-responsive element (ABRE) and the JA-responsive elements (CGTCA-motif and TGACG-motif), and some abiotic stress-responsive elements, such as the low-temperature-responsive element (LTR), the salicylic acid-responsive element (TCA-element) and the anaerobic-responsive element (ARE), were identified. Some over-presented CREs, including ARE, ABRE, CGTCA-motif, TGACG-motif, LTR and TC-rich repeats, were involved in the molecular response of plants to phytohormone, defense and stress responsiveness (Figure 2a). Among these, the MYB recognition site was most enriched, implying that the MYB transcript factors may play crucial roles in the transcriptional regulation of the *BnaNPF* genes. Besides, RY-element, the CRE involved in seed-specific regulation, was identified in the promoters of the 15 *BnaNPF* genes, which indicated that these *BnaNPF* genes might function in the process of seed development and matter storage.

### 2.4. Gene Expression Pattern Analysis of NPF Genes in Diverse Tissues of B. napus

**Figure 3:**
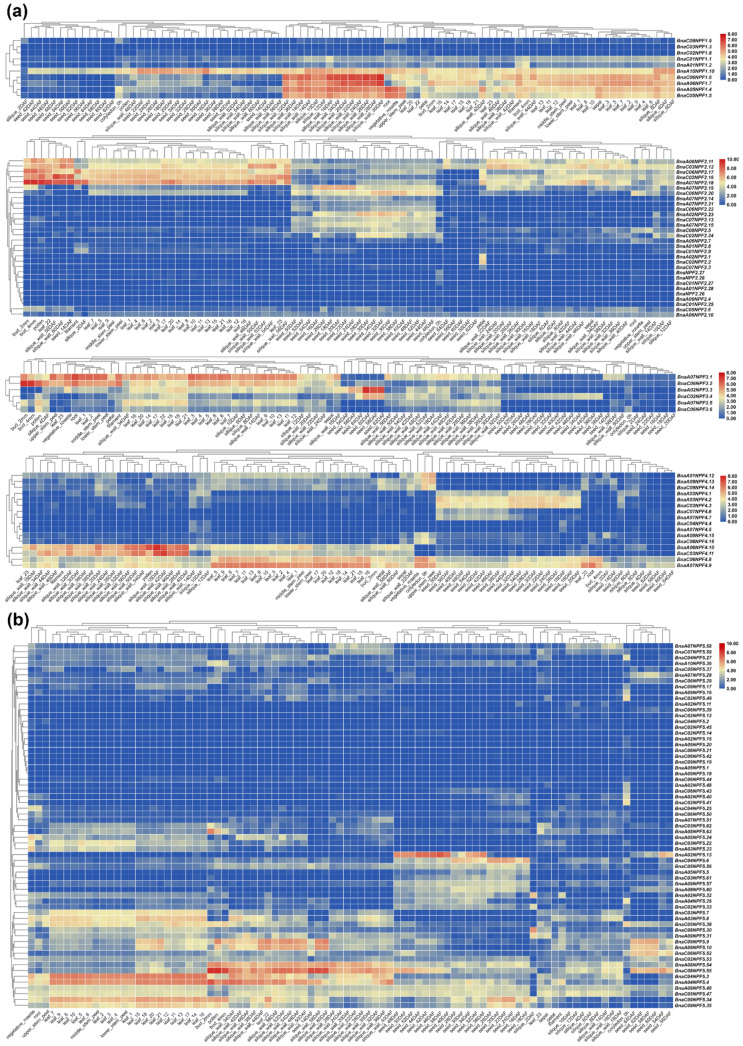

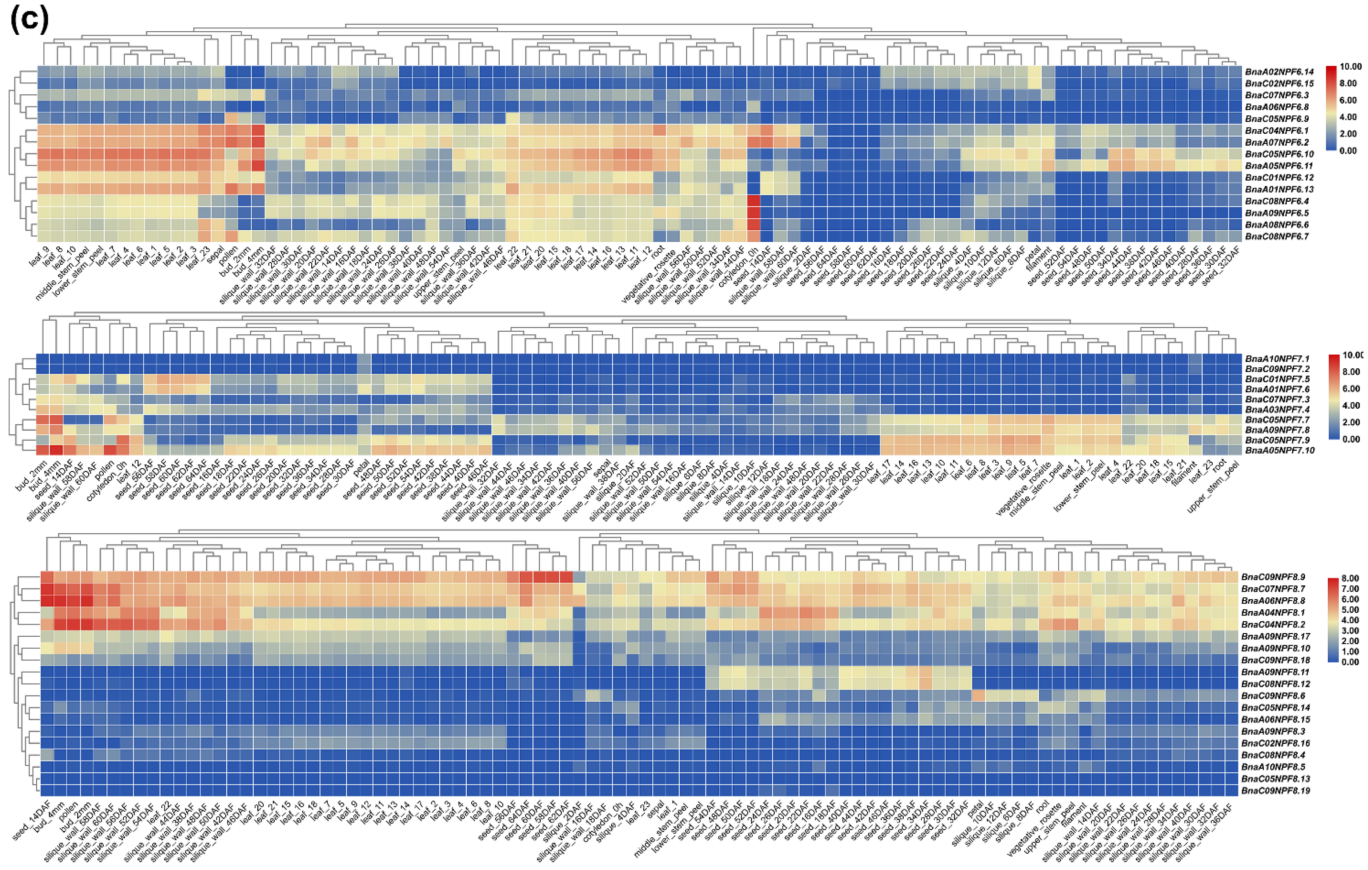
Gene expression profiles of *NPF* genes for 90 tissues or periods in *B. napus*. (**a**–**c**) display the gene expression profiles of *NPF1–4*, *NPF5*, and *NPF6–8* genes, respectively. The word “DAF” and the number before it mean the days after flowering. The number following “leaf” means leaf in different growth stages and parts of the plant, which originates from the online website (http://yanglab.hzau.edu.cn/BnTIR/eFP, Accessed on 4 May 2021).

In order to explore the potential tissues in which *NPF* genes function in *B. napus*, the expression profiles were characterized in 90 different organs or tissues, including cotyledon, root, vegetative rosette, stem peel (peel of upper, middle and lower stem), leaf (23 parts or periods), sepal, petal, filament, pollen, bud, silique wall (30 development periods) and seed (24 development periods) based on transcriptome information from BnTIR (http://yanglab.hzau.edu.cn/BnTIR/eFP, Accessed on 4 May 2021). Except for half of the genes in the *BnaNPF2*, *BnaNPF5* and *BnaNPF8* subfamily that has relatively low expression values (FPKM < 1) or no expression, most of the *BnaNPF* genes had preferential expression profiles in the 90 tissues (Figure 3). For instance, half of the *BnaNPF1* genes showed high expression levels in the silique wall at the early and middle development stages and in leaves of all parts; one-third of *BnaNPF2* genes (10/30) showed specific expression in the seeds at early and middle development stages; most of the *BnaNPF7* genes (8/10) were highly expressed in the bud, petal, pollen and seeds. In general, expression patterns were conserved in each clade within a subfamily, but were quite different across different subfamilies, suggesting the expression differentiation trend of this gene family. For instance, expression patterns of *BnaNPF2* and *BnaNPF4* subfamilies were classified into three conserved patterns that were consistent with the three major clades in these two subfamilies, and while the expression profile of the *BnaNPF3* genes was similar in this subfamily.

**Figure 4:**
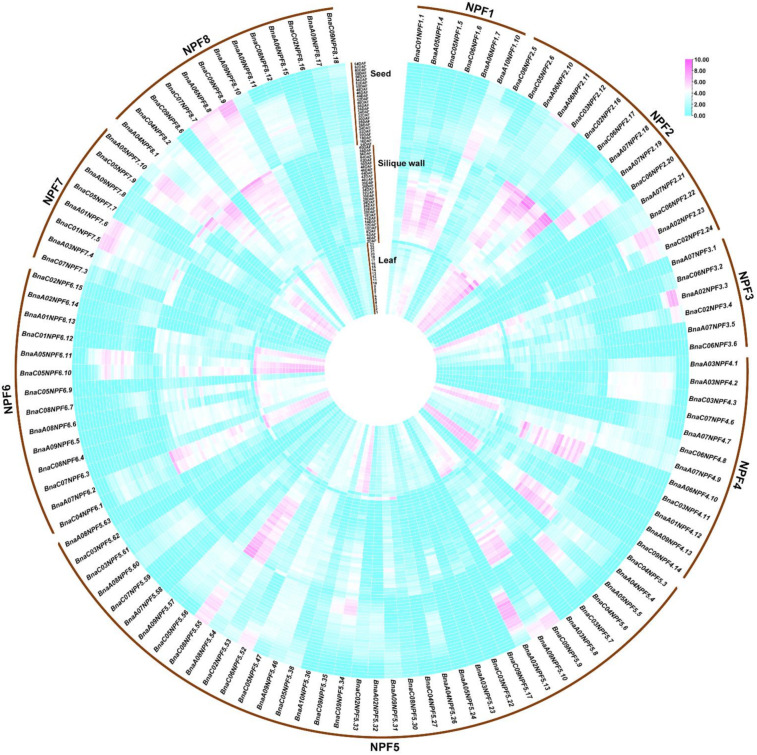
Expression changes of the *BnaNPF* genes at different development stages of leaves, silique wall and seeds. FPKM values were processed with log2 normalization on the column scale. The color scale represents relative expression levels from high (purple) to low (cyan).

Based on the expression profiles in seeds, silique wall and leaves from multiple development periods or plant parts, the expression patterns of *BnaNPF* genes in leaves, silique wall and seeds could be clarified clearly (Figure 4). Although some members of both *BnaNPF1* (4/10) and *BnaNPF2* (5/30) were highly expressed in the silique wall of the developing silique, *BnaNPF1* genes showed higher expression levels at the middle development stages, and *BnaNPF2* genes were higher expressed at the later development stages. The members of *BnaNPF3* with high expression levels in the silique wall, *BnaA07NPF3.1* and *BnaC06NPF3.2*, were higher expressed at the early than later development stage of the silique. However, some members of *BnaNPF4* and *BnaNPF5*, such as *BnaA06NPF4.10*, *BnaC03NPF4.11*, *BnaC04NPF5.3*, *BnaA04NPF5.4*, *BnaA08NPF5.54* and *BnaC08NPF5.55,* were higher expressed in silique wall at the later than early development stages of the silique. *BnaC09NPF5.9* and *BnaA09NPF5.10* were found preferential high expression in aged leaves and silique walls and nearly mature seeds. *BnaC01NPF7.5* and *BnaA01NPF7.6* showed higher expression at later development stages of seed, and *BnaC07NPF8.7*, *BnaA06NPF8.8* and *BnaC09NPF8.9* were preferentially higher expressed in aged leaves and silique wall.

### 2.5. Expression Dynamic of NPF Genes during the Growth of B. napus under Vernalization

**Figure 5:**
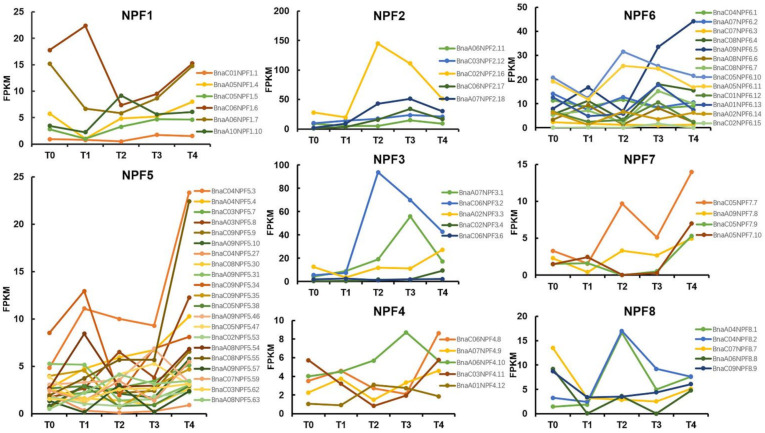
The expression changes of *BnaNPF* genes in leaves of ZS11 cultivar at five growth stages during vernalization. Plot FPKM value on the vertical *Y*-axis against growth stage on the horizontal *x*-axis. T0: 24 days post sowing and before vernalization; T1: 54 days post sowing and during vernalization; T2: 82 days post sowing and during vernalization; T3: 115 days post sowing and during vernalization; T4: 147 days post sowing and post vernalization.

There are differences in nutrition utilization and phytohormone distribution at different stages of plant growth. In order to explore the function and expression variation of *BnaNPF* genes, the expression trend of *BnaNPF* genes in leaves was analyzed during the growth of *B. napus* based on ZS11 transcription data from online data resources BnPIR (http://cbi.hzau.edu.cn/bnapus/, Accessed on 4 May 2021). Although the expression levels were quite different, members of the same subfamily usually have the same expression trend in leaves during the growth of *B. napus* (Figure 5). The members in subfamily *NPF*1, *NPF*4, *NPF*5 and *NPF*7 seem to be the same expression trend, decline at beginning of vernalization or in the early stage of vernalization and rise after vernalization. For example, *BnaC06NPF*1.6, as an ortholog of *AtNPF1.2* that was able to transport GA and JA, has the exact same expression trend and high expression level with *BnaC04NPF5.3* (homologous with *AtNPF5.1* that was able to transport GA, JA and ABA), which indicated that they might play important roles in phytohormone transport for a developmental phase transition. Some other members in *NPF*2, *NPF*3 and *NPF*6 shared this similar expression trend—that is, the expression level rising during vernalization and declining after vernalization. In typical cases, the expression level of *BnaC02NPF2.6*, *BnaC06NPF3.2* and *BnaC05NPF6.10* are dramatically raised from T1 to T2, and then begin to decline, which indicated that these members played an important role in the development stage during vernalization. Many *BnaNPF* genes showed diverse expression levels in the leaves of different cultivars at certain development stages (Figure S4). For example, *BnaA05NPF1.4* and *BnaC05NPF1.5* have no expression or lower expression levels in Shengli than other cultivars at T3 and T4 stages. At the T2 stage, *BnaC02NPF2.16* showed obviously a higher expression level in cultivars Quinta, Shengli and Tapidor than others. *BnaA06NPF8.8* has almost no expression during the whole development process in the three cultivars Shengli, Tapidor and Westar in comparison to other cultivars. These expression variations might lead to differences in nitrogen utilization efficiency, peptide transport and polar transport of phytohormone among the cultivars.

### 2.6. Transcriptional Analysis of BnaNPF Genes under Nitrate Deficiency

**Figure 6:**
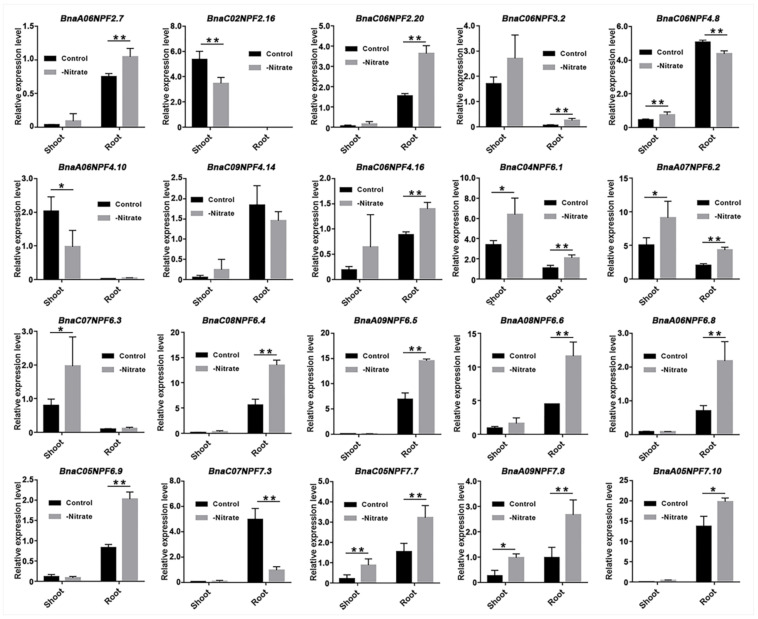
The expression changes detected for 20 *BnaNPF* genes under the condition of nitrogen suitability and deficiency. “Control” and “-Nitrate” represent treatments under nitrogen suitability and deficiency, respectively. “*”and “**” represent the significance level of 0.05 and 0.01, respectively.

Nitrate is the main substrate that *NPF* proteins transport, and more than one-third of *NPF* members have been reported to have a nitrate transport function in *Arabidopsis* [8]. Here, we analyzed the expression changes of *BnaNPF* genes under the condition of nitrogen suitability and deficiency. A total of 20 *BnaNPF* genes were detected to have relatively high expression and showed significant expression changes in shoot and/or root (Figure 6). Among them, six *BnaNPF* genes (*BnaC06NPF4.16*, *BnaC04NPF6.1*, *BnaA07NPF6.2*, *BnaA08NPF6.6*, *BnaC05NPF7.7* and *BnaA09NPF7.8*) were expressed at a high level in both shoot and root, and the expression levels were significantly elevated in both shoot and root after being treated with low nitrogen. Ten *BnaNPF* genes were specifically expressed in root, of which seven (*BnaA06NPF2.7*, *BnaC06NPF2.20*, *BnaC08NPF6.4*, *BnaA09NPF6.5*, *BnaA06NPF6.8*, *BnaC05NPF6.9* and *BnaA05NPF7.10*) were induced to highly express after low nitrogen treatment, which suggested they have a positive function for nitrogen absorption by roots. However, the expressions of the other three *BnaNPF* genes (*BnaC06NPF4.8*, *BnaC09NPF4.14* and *BnaC07NPF7.3*) that specifically expressed in roots were declined under low nitrogen treatment. In addition, four *BnaNPF* genes that were specifically expressed in shoots also showed different expression changes under low nitrogen treatment: *BnaC02NPF2.16* and *BnaA06NPF4.10* were upregulated, and the other two (*BnaC06NPF3.2* and *BnaC07NPF6.3*) declined after treated by low nitrogen.

## 3. Discussion

Although its genome is not the largest in comparison to the genomes of 33 plant species displayed in our study, *B. napus* contained the most *NPF* genes (Table 1). A total of 169 *BnaNPF* genes coding 186 proteins were identified in the *B. napus* genome in this study and designated as *BnaNPF1.1* to *BnaNPF8.19* in eight subfamilies based on phylogenetic analysis, and they exhibited evolutionary and syntenic relationships with *NPF* genes in *Arabidopsis*, *B. rapa,* and *B. olereaca*. Furthermore, the expression profiles of *BnaNPF* genes in 90 diverse tissues, as well as expression changes at different development stages under vernalization between eight rapeseed cultivars and under nitrate deficiency, were determined. This study provides a piece of basic information for further functional characterization of *BnaNPF* genes in the growth and development of *B. napus*. Recently, in apple and wheat [13,39], the NPF protein family was characterized and also defined as eight subfamilies, and according to eight subfamilies of *NPF* defined in these species and phylogenetic analysis, the 169 *BnaNPF* genes were identified and classified into eight unambiguous subfamilies, and BnaNPF subfamilies showed similar member proportions with these in *Arabidopsis* and wheat [1,13]. Recently, the identification of NPF genes had been reported in *B. napus*; 193 and 199 *BnaNPF* genes were identified in two studies, respectively [45,46]. Here, in order to excavate functional NPF, we sifted out the candidate NPF protein with less than 200 amino acid residues and 20% of the PTR2 domain missing, and only 169 NPF genes encoding 186 potential functional proteins were obtained for rapeseed finally. The 169 NPF proteins have a relatively complete protein sequence and PTR2 domain, and were likely to be functional and able to transport of substrates including amino acids, nitrate, phytohormones and glucosinolates. Brassicaceae species experienced a common whole genome soon after divergence from the *Arabidopsis* lineage approximately 17 to 20 million years ago [40,48]. *B. napus* is an allotetraploid (AnAnCnCn) that evolved from a spontaneous hybridization event between *B. rape* (ArAr) and *B. oleracea* (CoCo) about 7500 years ago [42], and has suffered a whole-genome triplication and a hybridization event compared with *Arabidopsis*. In theory, there should be three times as much NPF genes in *B. rapa* and *B. oleracea* (53 × 3), and six times as much *NPF* genes in *B. napus* (53 × 3 × 2) as in *Arabidopsis*. Fifty-three *NPF* members were identified in the *Arabidopsis* genome, it was expected that *NPF* genes may be expanded to about 160 genes in *B. rapa* or *B. oleracea*, and about 320 genes in *B. napus* genomes, respectively. However, only 82, 70 and 169 *BnaNPF* genes were identified in these three species, respectively, in this study (Table 1), which revealed that genome replication was accompanied by a large-scale loss of genes during evolution that was identical to the previous reports [49,50]. The A sub-genome and C sub-genome of *B. napus* (AACC) originated from *B. rapa* (AA) and *B. oleracea* (CC), respectively. Compared to both ancestral species, fewer *NPF* genes (76) were identified in the A sub-genome and more *NPF* genes (93) were discovered in the C sub-genome of *B. napus*. *NPF* genes in the C sub-genome were amplified obviously, which happened probably due to chromosome rearrangement or gene replication when *B. napus* formed by hybridization between *B. rapa* and *B. oleracea* about 7500 years ago [42]. Besides, *NPF* genes distributed on *B. rapa* (88.16%) and *B. oleracea* (55.91%) genome keep good collinearity with *NPF* genes on the A and C sub-genome of *B. napus*, respectively (Figure 1). These results indicate that *BnaNPF* genes have undergone not only chromosome segment replication, but complex recombination and gene loss in evolution processes, which is consistent with the recent reports [45,46].

The function and expression level of a gene is usually closely related to its gene characteristic and CREs [51]. Therefore, *BnaNPF* genes were further characterized for gene structures, protein conserved domains and CREs in this study. Most of the *BnaNPF* genes exhibited relatively concentrated distributional property in gene length (246–1568 bp) and amino acid number (400–600). In gene structure, most of the *BnaNPF* genes contained at most six extrons, and different *BnaNPF* subfamily genes exhibited significant exon–intron structural divergences, but *BnaNPF* within the same branches share similar gene structures, motifs, and localization patterns. Besides, CREs involved in hormone responses and MYB recognition site were detected in the promoter region of *BnaNPF* genes except for the common CREs, which indicated the expression of *BnaNPF* genes regulated by phytohormones and secondary metabolites (Figure 2).

Gene expression patterns provided imperative clues to map out gene functionality. In this study, the expression level of *BnaNPF* genes was investigated in diverse tissues or organs of *B. napus* using the released transcriptome information resource (http://yanglab.hzau.edu.cn/BnTIR/eFP, Accessed on 4 May 2021). Gene expression analysis showed that *BnaNPF* genes have significantly different and complex expression patterns across different subfamilies, but the expression pattern was conserved in each clade within a subfamily, which reflected structure and function uniform (Figure 3). Some *BnaNPF* genes showed obvious tissue preferential expression. Half of the *BnaNPF1* genes having ultrahigh expression levels in silique wall at the early and middle development stages of silique indicated efficient nitrogen transport for nutrient synthesis in the seed. *BnaA07NPF2.18*, orthologous to *AtNPF2.13/NRT1.7* that was able to transport nitrate, glucosinolates, JAs and GAs [14,52], was expressed at an ultrahigh expression level in the bud, pollen, filament and petal, which contribute to the previous reports that nitrate and nitrogen regulated flowering, and high nitrate/nitrogen helped promote flowering [53], and phytohormone played an important role in the regulation of flower organogenesis [54,55].

NPF proteins can transport a huge variety of substrates, including dipeptides, nitrate, glucosinolates, amino acids and several plant hormones [8], and the complex gene expression pattern would endow *BnaNPF* versatile roles in the growth and development of *B. napus*. Many *BnaNPF* genes were found to have a changing expression in the development of leaf, silique wall and seed that played a key role in yield (Figure 4). For example, in the *BnaNPF2* subfamily, *BnaA06NPF2.10*, *BnaA06NPF2.11* and *BnaC03NPF2.12* were orthologs to *AtNPF2.10*, and *BnaC02NPF2.16, BnaC06NPF2.17* and *BnaA07NPF2.18* were orthologs to *AtNPF2.13*, of which two *Arabidopsis* orthologs had the function of transporting glucosinolates [11,56] and showed up-regulated expression in the later stages of the silique and seed development of *B. napus*. Many CREs involved in hormone responses were detected in the promoter region of *BnaNPF* genes, including IAA- (103/169 genes), ABA- (123/169 genes), and MeJA-responsive CRE (119/169 genes) (Figure 2 and Table S4), which suggested their potential hormone-inducing characteristics. The process of plant growth and development was regulated by phytohormone directly, which might be why the transcription of *BnaNPF* genes was regulated responding to growth and development. Besides, with the development of plant organs, secondary metabolite accumulation level perhaps also played a part in the expression changes of some *BnaNPF* genes because of the existence of the MYB recognition site in their promoters [57].

The expression changes of *BnaNPF* genes during the growth of *B. napus* under natural vernalization were analyzed in this study. Vernalization is an important process that regulates the transition from vegetative growth to reproduction in *B. napus* [58,59], and involved in the regulation of various environmental factors and hormones [60]. The *BnaNPF* genes that expressed in leaves exhibited two expression trends: the first one, decline at the beginning of vernalization or in the early stage of vernalization and a rise after vernalization (most of the members of the *NPF*1, *NPF*4, *NPF*5 and *NPF*7 subfamily); the second one, the expression level was raised during vernalization and declined after vernalization (most of the members of the *NPF*2, *NPF*3 and *NPF*6 subfamily) (Figure 5). These results indicate that many *BnaNPF* genes might participate in floral transition and play different roles in the reproduction and development of *B. napus*. Based on the transcriptome data of eight cultivars from the BnPIR database, significant expression variation was found for some *BnaNPF* genes in different cultivars (Figure S4). These expression variations might lead to differences in transport of the corresponding substrates among the cultivars, which is expected for further functional research in the future.

Nitrate and phytohormone signaling pathways crosstalk to modulate growth and developmental programs in a multifactorial manner [61]. So far, more than half of the functionally characterized *NPF* genes have been demonstrated to be able to transport nitrate in *Arabidopsis* [13]. Here, twenty *BnaNPF* genes, ortholog to 11 *AtNPF* genes, were detected to respond to low nitrate treatment (Figure 6). The six members of the *BnaNPF*6 subfamily, *BnaNPF6.4–6.9* orthologous with *AtNPF6.3/NRT1.1*, were predominantly expressed in roots and were significantly up-regulated under low nitrogen treatment, suggesting their functional importance in nitrogen utilization efficiency. *AtNPF6.3* is the first plant *NPF* member that is characterized for functioning in nitrate uptake in the root, root-to-shoot transport and transceptor in sensing/signaling, and govern many molecular, physiological, and morphological responses to nitrate [6,15,16]. The gene expansion and consistent expression patterns in *B. napus* indicated the function uniformity of *NPF6.3* orthologs in nitrogen utilization efficiency as previously reported [62,63]. As the ortholog of *AtNPF2.9/NRT1.9* has been reported to facilitate the loading of nitrate into the root phloem and enhance downward nitrate transport in roots [64], *BnaA06NPF2.7* was up-regulated significantly in root under low nitrate conditions, while its homolog in rice, *OsNPF2.4*, was discovered by a genome-wide association study (GWAS) on nitrogen utilization efficiency-related agronomic traits [65]. So, *BnaA06NPF2.7* might also play an important role in nitrate transport in roots in *B. napus*, which needs to be characterized in the future. In addition to the role as a nutrient, nitrate acts as a signal molecular, and N nutrition and plant hormone signaling pathways are closely interconnected [61]. *BnaC02NPF2.16* and *BnaC06NPF3.2*, orthologues with *AtNPF2.13* and *AtNPF3.1*, respectively, were predominantly expressed in leaves. According to previous reports regarding *AtNPF2.13* and *AtNPF3.1*, remobilizing nitrate from old to young leaves involved GA accumulation and responses [66,67,68]. *BnaC02NPF2.16* and *BnaC06NPF3.2* might function in nitrite accumulation in leaves coupling hormone signal, which may be possible but needs to be verified in the future.

In this study, we provided a comprehensive understanding of the evolution and expression characteristics of *BnaNPF* genes in *B. napus*. It gives an important implication for further understanding the biological functions of individual *BnaNPF* genes. However, the study only provided a preliminary characterization of *BnaNPF* genes, and large functional validation work needs to be done in further work to understand the roles of *BnaNPF* genes.

## 4. Materials and Methods

### 4.1. Data Resource Related to NPF Gene Acquisition

The 53 *NPF* protein sequences from *Arabidopsis* were used as query, and a BLASTp search (E-value < 10^−10^) was performed to identify *NPF* genes in *B. rapa*, *B. oleracea*, *B. napus* and other plant species through the online BLAST tool in the databases, including The *Arabidopsis* Information Resource (TAIR, https://www.Arabidopsis.org/, Genome Version Araport11, Accessed on 4 May 2021), the Brassica Database (BRAD, http://brassicadb.org/brad/, var. ‘Chiifu’ and ‘TO1000′, Accessed on 4 May 2021 ), *Brassica napus* Pan-Genome Information Resource (BnPIR, http://cbi.hzau.edu.cn/bnapus/, var. ZS11, Accessed on 4 May 2021), and National Center for Biotechnology Information (NCBI, https://blast.ncbi.nlm.nih.gov/Blast.cgi, Accessed on 4 May 2021). Then, the potential *NPF* proteins were confirmed through the hidden Markov model (HMM) search program (HMMER v3.0, http://hmmer.janelia.org/, Accessed on 4 May 2021) with E-value below e-200, and the conserved domain database (CDD) (http://www.ncbi.nlm.nih.gov/Structure/bwrpsb/bwrpsb.cgi, Accessed on 4 May 2021) based on the presence of the PTR2 domains (PF00854). Furthermore, the candidate *NPF* protein of *B. rapa*, *B. oleracea* and *B. napus* with less than 200 amino acid residues and 20% of PTR2 domains missing was removed, and the rest were thought to be considered to be functional and used for further analysis.

### 4.2. Multiple Sequence Alignment and BnaNPF Genes Nomenclature

Full-length sequences of the *NPF* proteins from *Arabidopsis* and three *Brassica* crops were aligned with ClustalW, and then these alignments were used to construct the phylogenetic trees by the software MEGA Version 10.1.0 [69] with the neighbor-joining method. P-distance, pairwise deletion, and bootstrapping (1000 replicates) were set as the required parameters.

*NPF* genes were named according to the nomenclature Leran et al. (2014) recommended [1]. According to eight subfamilies of *NPF* in *Arabidopsis* and phylogenetic relationships, the clade number of *NPF* members would be ensured. Then, *NPF* members were named with two or three letters to identify the species, followed by “*NPF* + clade number (followed by a point) + the order number (which they are identified in phylogenetic tree)”, for instance, “*BraNPF2.3*”. Consequently, this second number is used to differentiate genes within the species and does not reflect orthologous relationships. The *NPF* members from *B. napus* obeyed the nomenclature convention but modified with adding chromosome between species name and “*NPF*”. If multiple *NPF* proteins were translated from the transcripts of the same gene, they were distinguished by the English letters “a”, “b” and “c”.

### 4.3. Chromosomal Location and Syntenic Analysis

The genomic locations of all *BnaNPF* genes were mapped to chromosomes of the *B. napus* genome according to the reference genome information of ZS11 in the BnPIR database. The synteny orthologous gene pairs were identified based on BLASTP (identity > 75%, and E-value < 10^−10^) and phylogenetic relationship. The chromosomal regions within 200 kb containing a string of two or more genes were defined as tandem duplication [70]. The nonsynonymous rate (Ka), synonymous rate (Ks), and Ka/Ks between the orthologous gene pairs were calculated using the NY method implemented in the Ka/Ks calculator program [71] according to gene CDS pairwise alignment performed with Clustal W (https://www.genome.jp/tools-bin/clustalw, Accessed on 4 May 2021).

### 4.4. Functional Domain Validation and Cis-Acting Regulatory Elements (CREs) Prediction

The protein sequence and full-length coding sequences (CDS) information of the At*NPF*s and *BnaNPF*s were retrieved and extracted from the *Arabidopsis* Information Resource (TAIR: https://www.Arabidopsis.org/index.jsp, Accessed on 4 May 2021) and *Brassica napus* pan-genome information resource (BnPIR: http://cbi.hzau.edu.cn/bnapus/index.php, Accessed on 4 May 2021). To examine the structural divergence among the NPF proteins in *Arabidopsis* and *B. napus*, the protein sequences were subjected to the HMMER software (http://www.ebi.ac.uk/Tools/hmmer, Accessed on 4 May 2021) to predict and characterize the conserved domains with default parameters. A 2.0 kb genomic sequence upstream from the start codon was downloaded for each gene from the BnPIR website (http://cbi.hzau.edu.cn/bnapus/index.php, Accessed on 4 May 2021). These sequences were subjected to plantCARE (http://bioinformatics.psb.ugent.be/webtools/plantcare/html/, Accessed on 4 May 2021) to identify putative CREs, and CRE distribution in the promoter region was displayed by TBtools software [72]. The Gene Structure Display Server (GSDS Version 2.0, http://gsds.cbi.pku.edu.cn/index.php, Accessed on 4 May 2021) was used to display the exon–intron structures of the *NPF*s in *Arabidopsis* and *B. napus*.

### 4.5. Identification of the Expression Pattern of BnaNPF Genes in B. napus

The fragments per kilobase of exon model per million mapped fragments (FPKM) value of 169 *BnaNPF* genes in different organs or tissues, including cotyledon, root, vegetative rosette, stem peel (peel of upper, middle and lower stem), leaves (23 parts or periods), sepal, petal, filament, pollen, bud, silique wall (30 development periods) and seeds (24 development periods) based on transcriptome information, were retrieved from BnTIR (http://yanglab.hzau.edu.cn/BnTIR/eFP, Accessed on 4 May 2021). RNA-seq for leaves of all eight rapeseed accessions at five stages with a one-month interval derived from BnPIR (http://cbi.hzau.edu.cn/bnapus/expression/, Accessed on 4 May 2021), including one stage before vernalization (T0), three stages during vernalization (T1, T2 and T3), and one stage post vernalization (T4), was used to analyze *BnaNPF* gene expression patterns at different development stages.

### 4.6. Expression Analysis of BnaNPF Genes under Low Nitrate Stress

To further investigate the transcriptional responses of *BnaNPF* genes under low nitrate stress, the uniform *B. napus* seedlings (var. ZS11) were hydroponically cultured in Hoagland nutrient solution for 10 days at 7 days after seed germination, and then parts of them were transferred to Hoagland nutrient solution modified with low nitrate (0.3 mM NO^3−^) for 3 days. The rapeseed seedlings were cultivated in the culture room as Cui et al. (2020) described [73]. The shoots and roots under low nitrogen treatment for 72 h and control were individually harvested and immediately stored at −80 °C for RNA isolation, and each sample contained 3 independent biological replicates. Total RNA was isolated from the frozen samples using a RNAprep Pure Plant Kit (Tiangen), and first-strand cDNA was synthesized from the total RNA using a PrimeScriptTM RT Master Mix Kit (TaKaRa). The cDNA was subjected to quantitative analysis using SYBR^®^ Premix Ex Taq^TM^ (Takara Bio, Shiga, Japan) on the Applied Biosystems StepOne^TM^ Plus Real-time PCR System (Thermo Fisher Scientific, Waltham, MA, USA), as previously described [73]. The *BnaNPF* primer sequences were obtained from the qPCR Primer Database [74] and are listed in Table S1. The housekeeping gene *BnaACTIN7* was used as a reference gene for normalization and to analyze the *BnaNPF* gene expression levels via the 2^−ΔΔCt^ method. Three independent technical replicates were performed for each sample.

## 5. Conclusions

A total of 169 *NPF* gene members were identified in the *B. napus* genome and classified into eight subfamilies in this study. The *BnaNPF* genes were unevenly distributed in the *B. napus* genome and exhibited obvious synteny and orthologous duplication with *NPF* genes in *Arabidopsis*, *B. rapa* and *B. olereaca*. Moreover, the complex expression patterns of *NPF* genes in various tissues and periods were investigated, and the expression changes at different development stages under nature vernalization and response to nitrate deficiency were determined in *B. napus*. The evolution and expression pattern analysis of *NPF* genes will provide valuable information for further functional characterization in rapeseed.

## Figures and Tables

**Table 1:** The synteny relationship of *NPF* genes between *B. rapa* and *B. napus*, and between *B. oleracea* and *B. napus.*

Ratio ^a^	0:1	1:0	1:1	1:2	1:3	1:5
***B. rapa***	2	9	55	16	1	1
***B. oleracea***	4	9	54	7		

^a^ Orthologous NPF gene ratio by comparing *B. rapa* and *B. oleracea* with the *B. napus* genome. 0:1 represents NPF orthologs lost in the *B. rapa* or *B. oleracea* genome, 1:0 represents NPF orthologs lost in the *B. napus* genome, and 1:2, 1:3 and 1:5 represent different replication multiples in the *B. napus* genome.

**Table 2:** Copy number variations (CNVs) of the *NPF* genes in 36 plant species.

Organism Name	*NPF*1	*NPF*2	*NPF*3	*NPF*4	*NPF*5	*NPF*6	*NPF*7	*NPF*8	Total	Genome Size (Mb)
***Arabidopsis*** lyrata (D)	3	14	1	9	17	4	3	5	56	202.97
*Arabidopsis thaliana* (D)	3	14	1	7	16	4	3	5	53	127.42
*Aquilaria agallochum* (D)	6	7	3	12	13	5	3	6	55	726.71
*Brachypodium distachyon* (M)	2	6	4	13	21	8	11	17	82	271.3
***Brassica rapa*** (D)	4	23	3	9	23	7	5	8	82	401.93
***Brassica oleracea*** (D)	4	15	2	8	26	6	5	4	70	554.98
***Brassica napus*** (D)	10	30	6	16	63	15	10	19	169	976.19
*Carica papaya* (D)	4	14	3	8	12	8	6	4	59	370.42
*Capsella rubella* (D)	3	12	1	6	17	4	3	5	51	133.06
*Citrus clementina* (D)	9	7	3	9	17	6	4	4	59	301.37
*Citrus sinensis* (D)	8	7	3	10	17	6	4	4	59	319.23
*Cuscuta campestris* (D)	4	9	3	8	19	6	5	5	59	476.79
*Eucalyptus grandis* (D)	6	12	4	11	19	6	4	6	68	691.43
*Fragaria vesca* (D)	0	13	2	8	23	3	5	6	60	214.37
*Glycine max* (D)	13	14	6	22	41	11	14	13	134	927.71
*Gossypium raimondii* (D)	7	10	4	14	14	11	7	8	75	773.77
*Linum usitatissimum* (D)	12	7	4	14	25	9	11	10	92	316.17
*Malus domestica* (D)	2	34	4	21	44	17	8	9	139	1874.77
*Manihot esculenta* (D)	7	12	6	10	23	7	5	5	75	292.1
*Medicago truncatula* (D)	8	12	3	14	25	8	9	1	80	412.92
*Oryza sativa* (M)	3	6	5	12	29	6	11	21	93	389.75
*Phaseolus vulgaris* (D)	8	11	3	12	22	5	7	6	74	521.08
*Populus trichocarpa* (D)	15	9	5	12	26	6	5	7	85	434.29
*Prunus persica* (D)	2	15	1	8	16	5	5	5	57	214.22
*Ricinus communis* (D)	5	20	3	7	13	5	4	3	60	350.62
*Setaria italica* (M)	4	11	8	16	19	7	12	21	98	405.87
*Solanum tuberosum* (D)	17	10	2	15	8	9	4	8	73	772.25
*Solanum lycopersicum* (D)	19	16	2	12	11	12	7	11	90	760.07
*Sorghum bicolor* (M)	4	8	7	16	22	6	9	19	91	709.35
*Theobroma cacao* (D)	4	14	3	10	19	7	4	5	66	345.99
*Vitis vinifera* (D)	4	7	2	6	21	5	4	3	52	486.2
*Zea mays* (M)	4	4	6	12	17	8	12	16	79	2271.03
*Amborella trichopoda* (D)	1	5	2	7	15	4	3	7	45	706.60
*Physcomitrella patens* (L)	0	0	1	1	8	6	3	4	23	472.081
*Selaginella moellendorffii* (L)	0	0	4	4	11	6	5	16	46	212.315
*Selaginella moellendorffii* (L)	0	0	4	4	11	6	5	16	46	212.315

D dicots, M monocots, L lower plants.

## Data Availability

Not applicable

## Supplementary Materials

The following are available online at https://www.mdpi.com/article/10.3390/ijms22094944/s1, Figure S1: Phylogenetic relationships of the *NPF* proteins in *Arabidopsis* and *B. napus*. Neighbour-joining bootstrap values are shown above the branch, the branches of different subfamilies are colored differently, and the terminals of the branch labeled red represent the *Arabidopsis NPF* proteins. Figure S2: The synteny of *NPF* genes among *Arabidopsis*, *B. rapa*, *B. oleracea* and *B. napus*. Figure S3: Gene structure and protein conserved domain architecture of the *BnaNPF* genes. On the left is the gene structure and on the right is the protein conserved domain architecture. *NPF1* and *NPF2* clustered in a branch were displayed in (a), *NPF3*, *NPF4* and *NPF6* were displayed in (b), *NPF5* was displayed in (c), and *NPF7* and *NPF8* clustered in a branch were displayed in (d). Figure S4: The expression diversity of *BnaNPF* genes in leaves of eight cultivars at five growth stages during vernalization. Table S1: The primer sequences for the qRT-PCR. Table S2: Ka, Ks and Ka/Ks of orthologous pairs on *BnaNPF* and *AtNPF* genes. Table S3: The synteny relationship of NPF genes between *B. napu*s and *B. rape* and *B. oleracea*. Table S4: The CREs detected in the promoter of the *BnaNPF* genes.
